# Should patients with minor strokes be given thrombolytics?

**DOI:** 10.1136/svn-2024-003451

**Published:** 2024-08-03

**Authors:** Xun Wang, Yi Dong, Qiang Dong, David Wang

**Affiliations:** 1 Department of Neurology, Huashan Hospital, State Key Laboratory of Medical Neurobiology, Fudan University, Shanghai, China; 2 Neurovascular Division, Department of Neurology, Barrow Neurological Institute, Phoenix, Arizona, USA

**Keywords:** Stroke, Thrombolysis, Asprin, Ischemic Stroke

## Abstract

Mild stroke symptoms are cited as the reason for not using tissue-type plasminogen activator in 29–43% of time-eligible patients. Previous studies suggested that not all of these patients had a good recovery or even survival to hospital discharge. Since then, stroke guidelines worldwide recommended thrombolysis in minor but disabling strokes.

Dual antiplatelet treatment with aspirin and clopidogrel was more effective than aspirin alone for reducing subsequent events in patients with minor stroke if started within 24 hours of onset in both CHANCE (Clopidogrel in High-Risk Patients with Acute Non-disabling Cerebrovascular Events) and POINT (Platelet-Oriented Inhibition in New TIA and Minor Ischaemic Stroke) trials. Recently, both PRISMS (The Potential of rtPA for Ischemic Strokes With Mild Symptoms) trial and TEMPO-2 (Tenecteplase Versus Standard of Care for Minor Ischemic Stroke With Proven Occlusion) trial showed that treatment with thrombolysis versus antiplatelet did not increase the likelihood of favourable functional outcome at 90 days among patients with minor non-disabling acute ischaemic strokes. Therefore, a narrative review on thrombolysis for patients with minor strokes from published studies may help practicing clinicians.

## Introduction

 Mild stroke symptoms are cited as the reason for not using tissue-type plasminogen activator in 29–43% of time-eligible patients.^1 2^ Previous studies suggested that not all of these patients had a good recovery or even survive to hospital discharge.^3^,^6^ A large nationwide study (Get With The Guidelines–Stroke) showed that stroke-related disability in mild stroke is relatively common. They also illustrated the clinical outcomes at discharge were strongly associated with the initial NIHSS(National Institutes of Health Stroke Scale) scores.^7^ The multinational Safe Implementation of Treatment in Stroke-International Stroke Thrombolysis Registry observational study showed patients with a minor stroke had 71–72% favourable outcome (modified Rankin Scale, mRS 0–1) at 3 months, regardless of the time window of presentation.^8^ Since then, stroke guidelines worldwide recommended thrombolysis in minor but disabling strokes.^9^,^12^

Dual antiplatelet treatment with aspirin and clopidogrel were more effective than aspirin alone for reducing subsequent events in patients with minor stroke if started within 24 hours of onset in both CHANCE (Clopidogrel in High-Risk Patients with Acute Non-disabling Cerebrovascular Events) and POINT (Platelet-Oriented Inhibition in New TIA and Minor Ischaemic Stroke) trials.^13 14^ Recently, both PRISMS (The Potential of rtPA for Ischemic Strokes With Mild Symptoms) and TEMPO-2 (Tenecteplase Versus Standard of Care for Minor Ischemic Stroke With Proven Occlusion) trials showed that treatment with thrombolysis versus antiplatelet did not increase the likelihood of favourable functional outcome at 90 days among patients with minor nondisabling acute ischaemic strokes.^15 16^ Therefore, a narrative review on thrombolysis for patients with minor strokes from published studies may help the practicing clinicians (table 1)，

**Table 1:** Different definitions of minor stroke in different studies

Definitions	Study
(A) All patients with a score 0 or 1 on every baseline NIHSS score item, except level of consciousness items (items 1a to 1c), which must be 0.	EXPRESS^27^
(B) All patients with a lacunar-like syndrome (presumed small-vessel occlusive disease) such as pure sensory syndrome, pure motor hemiparesis, sensorimotor syndrome, ataxic hemiparesis and dysarthria-clumsy hand syndrome.	TOAST^28^
(C) Baseline NIHSS in the lowest (least severe) quartile of severity (NIHSS≤9).	DATAS II^29^
(D) Baseline NIHSS≤3.	CHANCE,^13^ CHANCE 2,^30^ POINT^14^
(E) Baseline NIHSS 0–5.	SOCRATES,^31^ THALES,^32^ PRISMS,^15^ INSPIRES^16^
(F) Baseline NIHSS≤5, with 1 point on the NIHSS in several key single-item scores, such as vision, language, neglect or single limb weakness, and a score of 0 in the consciousness item.	ARAMIS^18^

ARAMIS, Antiplatelet vs R-tPA for Acute Mild Ischemic Stroke.; CHANCE 2, Clopidogrel with Aspirin in High-Risk Patients with Acute Nondisabling Cerebrovascular Events II; CHANCE, Clopidogrel in High-Risk Patients with Acute Non-disabling Cerebrovascular Events; DATAS II, Dabigatran Treatment of Acute Stroke II; EXPRESS, Early use of EXisting PREventive Strategies for Stroke; INSPIRES, Intensive Statin and Antiplatelet Therapy for Acute High-Risk Intracranial or Extracranial Atherosclerosis; NIHSS, National Institutes of Health Stroke Scale; POINT, Platelet-Oriented Inhibition in New TIA and Minor Ischemic Stroke; PRISMS, Potential of r-tPA for Ischemic Strokes With Mild Symptoms; SOCRATES, Acute Stroke or Transient Ischemic Attack Treated With Aspirin or Ticagrelor and Patient Outcomes; THALES, Acute Stroke or Transient Ischemic Attack Treated With Ticagrelor and ASA for Prevention of Stroke and Death; TOAST, Trial of Org 10172 in Acute Stroke Treatment .

## Different criteria of minor stroke

A NIHSS ≤3 or ≤5 were widely used to define a minor stroke, although the consensus is still lacking.^17^

## Impact of guidelines

Most published guidelines for acute ischaemic stroke suggest thrombolytic therapy to treat patients with a disabling minor ischaemic stroke within 4.5 hours, while most guidelines do not recommend thrombolysis in patients with non-disabling minor strokes. (Table 2).However, disabling stroke has not been defined well. It is also unclear what the best treatment is in patients with acute ischaemic stroke (AIS) low NIHSS but from a large vessel occlusion. Dual antiplatelet therapy could be an option for patients with AIS with an NIHSS<3 and given within 24 hours.

**Table 2:** Comparison of guidelines recommendation on minor stroke and level of evidence

Guidelines	Recommendation	COR/LOE
Chinese Stroke Association 2023	For patients with acute ischaemic stroke with mild and disabling symptoms within 4.5 hours of onset, intravenous thrombolysis is recommended.	IIa/B
	For patients with acute ischaemic stroke with mild non-disabling symptoms (NIHSS 0–5) within 4.5 hours, intravenous thrombolysis is not routinely recommended.	III/B
	For patients with minor ischaemic stroke and high-risk transient ischaemic attack who did not receive intravenous thrombolysis, dual antiplatelet therapy is initiated within 24 hours of symptom onset if their NIHSS score is <3.	I/A
	For patients with moderate ischaemic stroke (NIHSS score of 4–5) who present within 24 hours of symptom onset, ticagrelor plus aspirin for 30 days (ticagrelor loading dose of 180 mg on the first day, followed by 90 mg two times per day) may reduce the risk of recurrent stroke and death within 30 days.	IIb/B
American Stroke Association 2019	For otherwise eligible patients with mild stroke presenting in the 3-hour to 4.5-hour window, treatment with intravenous alteplase may be reasonable. Treatment risks should be weighed against possible benefits.	IIb/B
European Stroke Organisation (ESO) 2023	For patients with acute minor, disabling ischaemic stroke of <4.5-hour duration, we recommend intravenous thrombolysis with alteplase.	Moderate,strong
	For patients with acute minor non-disabling ischaemic stroke of <4.5-hour duration, we suggest no intravenous thrombolysis.	Moderate, weak
	For patients with acute minor non-disabling ischaemic stroke of <4.5-hour duration, and with proven large-vessel occlusion, there is insufficient evidence to make an evidence-based recommendation.	Very low
	For patients with acute minor non-disabling ischaemic stroke of <4.5-hour duration, and with proven large-vessel occlusion, there is insufficient evidence to make an evidence-based recommendation.	Expert consensus
	For patients with acute ischaemic stroke of <4.5-hour duration, and rapidly improving neurological signs, which are still disabling, there is insufficient evidence to make a recommendation.	Very low
	For patients with acute ischaemic stroke of <4.5-hour duration, and rapidly improving neurological signs, which are still disabling, intravenous thrombolysis with alteplase is recommended.	Expert consensus

COR, Classification of recommendation; LOE, Level of Evidence; NIHSS, National Institutes of Health Stroke Scale.

## The ceiling effects and floor effects

A ceiling effect associated with statistics in medical condition refers to the phenomenon in which the majority of the data are close to the upper limit or highest possible score of a test. This means that (almost) all of the test participants achieved the highest (or very near to the highest) score. Recently, PRISMS trial showed 122 patients (78.2%) in the alteplase group versus 128 (81.5%) in the aspirin group achieved a favourable outcome (adjusted risk difference, −1.1%; 95% CI, −9.4% to 7.3%) at 90 days.^15^ Additionally, ARAMIS (Antiplatelet vs R-tPA for Acute Mild Ischemic Stroke) trial demonstrated that at 90 days, 93.8% of patients (346/369) in the DAPT (dual antiplatelet treatment) group and 91.4% (320/350) in the alteplase group had an excellent functional outcome (risk difference, 2.3% (95% CI, −1.5% to 6.2%)).^18^ However, TEMPO-2 trial found 50% (226/452) in the control group and 58% (247/432) in the Tenecteplase group recovered to NIHSS 0 at discharge (RR 1.16, 95% CI, 1.01 to 1.31), while the difference became smaller at 90 days for favourable outcome (71% vs 69%, RR 0.97, 95% CI, 0.89 to 1.05).^16^ From these studies, we have learnt that the rate of 90-day mRS 0–1 in minor stroke was high, which might have already reached the ceiling effect. Since those ceiling effects can impact the quality of studies, an NIHSS of 0 at discharge might be more sensitive.

However, floor effects need to be considered as well. Floor effect is a phenomenon where participants’ scores are generally low and show no differences due to the high difficulty of the experiment. Among these three trials, the numbers of symptomatic intracerebral haemorrhage (sICH) were reported as 5 versus 0, 1 versus 3 and 2 versus 8 in each group, respectively.^15 16 18^ The haemorrhagic event rate was very low, which made the traditional comparative analytical method very limited.

One post-hoc analysis from the Alteplase Compared with Tenecteplase in Patients With Acute Ischaemic Stroke trial, the primary outcome (mRS score 0–1 at 90 days) among patients with minor stroke occurred in 100 participants (51.8%) in the tenecteplase group and 86 (47.5%) in the alteplase group. There were no significant differences in the rates of sICH (2.9% in tenecteplase vs 3.3% in alteplase group).^19^ Therefore, the safety and efficacy of thrombolysis in minor stroke from the real-world database might need to be further studied and promising.

## The rate of recurrent stroke and early neurological deterioration

Early neurological deterioration (END) occurs in about 10% of patients after intravenous thrombolysis (IVT) and is related to a poor outcome. In theory, early antiplatelet therapy following IVT could reduce END by preventing re-occlusion and stroke progression.^20^ However, current guidelines recommend starting antiplatelet treatment at 24 hours after IVT due to concerns of haemorrhagic transformation. Other antithrombotics studied including low molecular-weighted heparin, oral anticoagulation, intravenous tirofiban did not offer a definitive answer on their benefit and risks in preventing recurrent stroke or END.^20^,^22^ The ongoing Early Antiplatelet for Minor Stroke Following Thrombolysis trial may provide more information once completed.^23^

## Brain reperfusion and long-term mental health

In terms of mechanism of action, antiplatelet treatment is for secondary stroke prevention while thrombolysis is to open the occluded artery with brain reperfusion.^24^ Reperfusion may restore more brain function from strokes and preserve long-term mental health and less cognitive impairment.^25^ However, data to show the benefit of thrombolysis on post-stroke cognitive impairment is limited.

## Conclusion

Minor stroke is not minor.^26^ Thrombolysis or not for patients with minor stroke is in hot debate since more data have been published recently. These data suggested that DAPT might be as good as IVT in this group of patients. However, since the definition of a minor stroke may vary, and the long-term outcome such as END and cognition is unclear, selecting DAPT versus IVT remains a choice for the treating physician.

## References

[1] California Acute Stroke Pilot Registry (CASPR) Investigators (2005). Prioritizing interventions to improve rates of thrombolysis for ischemic stroke. Neurol (ECronicon).

[2] Kleindorfer D, Kissela B, Schneider A (2004). Eligibility for recombinant tissue plasminogen activator in acute ischemic stroke: a population-based study. Stroke.

[3] Smith EE, Abdullah AR, Petkovska I (2005). Poor outcomes in patients who do not receive intravenous tissue plasminogen activator because of mild or improving ischemic stroke. Stroke.

[4] Nedeltchev K, Schwegler B, Haefeli T (2007). Outcome of stroke with mild or rapidly improving symptoms. Stroke.

[5] Barber PA, Zhang J, Demchuk AM (2001). Why are stroke patients excluded from tpa therapy? An analysis of patient eligibility. Neurol (ECronicon).

[6] Rajajee V, Kidwell C, Starkman S (2006). Early MRI and outcomes of untreated patients with mild or improving ischemic stroke. Neurol (ECronicon).

[7] Smith EE, Fonarow GC, Reeves MJ (2011). Outcomes in mild or rapidly improving stroke not treated with intravenous recombinant tissue-type plasminogen activator: findings from get with the guidelines-stroke. Stroke.

[8] Ahmed N, Wahlgren N, Grond M (2010). Implementation and outcome of thrombolysis with alteplase 3-4.5 h after an acute stroke: an updated analysis from SITS-ISTR. Lancet Neurol.

[9] Liu L, Li Z, Zhou H (2023). Chinese Stroke Association guidelines for clinical management of ischaemic cerebrovascular diseases: executive summary and 2023 update. Stroke Vasc Neurol.

[10] Powers WJ, Rabinstein AA, Ackerson T (2019). Guidelines for the early management of patients with acute ischemic stroke: 2019 update to the 2018 guidelines for the early management of acute ischemic stroke: a guideline for healthcare professionals from the american heart association/american stroke association. Stroke.

[11] Berge E, Whiteley W, Audebert H (2021). European stroke organisation (ESO) guidelines on intravenous thrombolysis for acute ischaemic stroke. Eur Stroke J.

[12] Gladstone DJ, Lindsay MP, Douketis J (2022). Canadian stroke best practice recommendations: secondary prevention of stroke update 2020. Can J Neurol Sci.

[13] Wang Y, Wang Y, Zhao X (2013). Clopidogrel with aspirin in acute minor stroke or transient ischemic attack. N Engl J Med.

[14] Johnston SC, Easton JD, Farrant M (2018). Clopidogrel and aspirin in acute ischemic stroke and high-risk TIA. N Engl J Med.

[15] Khatri P, Kleindorfer DO, Devlin T (2018). Effect of alteplase vs aspirin on functional outcome for patients with acute ischemic stroke and minor nondisabling neurologic deficits: the PRISMS randomized clinical trial. JAMA.

[16] Coutts SB, Ankolekar S, Appireddy R (2024). Tenecteplase versus standard of care for minor ischaemic stroke with proven occlusion (TEMPO-2): a randomised, open label, phase 3 superiority trial. Lancet.

[17] Fischer U, Baumgartner A, Arnold M (2010). What is a minor stroke?. Stroke.

[18] Chen H-S, Cui Y, Zhou Z-H (2023). Dual antiplatelet therapy vs alteplase for patients with minor nondisabling acute ischemic stroke: the ARAMIS randomized clinical trial. JAMA.

[19] Nair R, Singh N, Kate M (2024). Intravenous tenecteplase compared with alteplase for minor ischaemic stroke: a secondary analysis of the AcT randomised clinical trial. Stroke Vasc Neurol.

[20] Gao Y, Pan Y, Han S (2023). Rationale and design of a randomised double-blind 2×2 factorial trial comparing the effect of a 3-month intensive statin and antiplatelet therapy for patients with acute mild ischaemic stroke or high-risk TIA with intracranial or extracranial atherosclerosis (INSPIRES). Stroke Vasc Neurol.

[21] Zi JS, Kong W (2023). Tirofiban for stroke without large or medium-sized vessel occlusion huang. N Engl J Med.

[22] Chen H-S, Cui Y, Zhou Z-H (2023). Effect of argatroban plus intravenous alteplase vs intravenous alteplase alone on neurologic function in patients with acute ischemic stroke. JAMA.

[23] Li X-Q, Cui Y, Wang X-H (2023). Early antiplatelet for minor stroke following thrombolysis (EAST): rationale and design. Int J Stroke.

[24] Ingleton A, Raseta M, Chung R-E (2024). Is intraprocedural intravenous aspirin safe for patients who require emergent extracranial stenting during mechanical thrombectomy?. Stroke Vasc Neurol.

[25] Wang Y, Li S, Zhou Q (2023). Vascular dementia has the highest hospitalisation rate in china: a nationwide hospital information system study. Stroke Vasc Neurol.

[26] Leng X, Wang D (2024). Minor stroke is not minor. Stroke Vasc Neurol.

[27] Rothwell PM, Giles MF, Chandratheva A (2007). Effect of urgent treatment of transient ischaemic attack and minor stroke on early recurrent stroke (EXPRESS study): a prospective population-based sequential comparison. The Lancet.

[28] Adams HP, Bendixen BH, Kappelle LJ (1993). Classification of subtype of acute ischemic stroke. Definitions for use in a multicenter clinical trial. TOAST. Trial of Org 10172 in Acute Stroke Treatment. Stroke.

[29] Butcher KS, Ng K, Sheridan P (2020). Dabigatran Treatment of Acute Noncardioembolic Ischemic Stroke. Stroke.

[30] Wang Y, Meng X, Wang A (2021). Ticagrelor versus Clopidogrel in *CYP2C19* Loss-of-Function Carriers with Stroke or TIA. N Engl J Med.

[31] Johnston SC, Amarenco P, Albers GW (2016). Ticagrelor versus Aspirin in Acute Stroke or Transient Ischemic Attack. N Engl J Med.

[32] Johnston SC, Amarenco P, Denison H (2020). Ticagrelor and Aspirin or Aspirin Alone in Acute Ischemic Stroke or TIA. N E J Med.

